# Acute dizziness in the emergency department: trends, diagnostic challenges and stroke mimics among thrombolysed patients

**DOI:** 10.3389/fneur.2026.1735022

**Published:** 2026-02-17

**Authors:** Thomas Bailey Tysland, Soffien Chadli Ajmi, Frederik Kragerud Goplen, Natascha Wathne, Mehdi Rezai, Øistein Rønneberg Mjelva, Martin W. Kurz

**Affiliations:** 1 Department of Neurology and Neuroscience Research Group, Stavanger University Hospital, Stavanger, Norway; 2 Department of Otorhinolaryngology and Head and Neck Surgery, Haukeland University Hospital, Bergen, Norway; 3 Department of Clinical Medicine, University of Bergen, Bergen, Norway; 4 Department of Otorhinolaryngology and Head and Neck Surgery, Stavanger University Hospital, Stavanger, Norway; 5 Department of Medicine, Stavanger University Hospital, Stavanger, Norway

**Keywords:** dizziness, emergency department, hints, stroke mimics, thrombolysis, vertigo

## Abstract

**Background:**

Acute dizziness is a frequent cause of emergency department (ED) visits, but differentiating benign vestibular disorders from posterior circulation stroke remains challenging. Neuroimaging has low sensitivity in the acute phase, while bedside tests such as HINTS has shown superior diagnostic accuracy when performed by trained providers. Efforts to reduce door-to-needle times (DNT) in thrombolysis may exacerbate diagnostic variability, increasing the risk of treating stroke mimics. This study aims to evaluate factors contributing to misdiagnosis by analyzing (i) temporal trends in total admissions and triage (ii) quality of clinical assessments and (iii) patterns in IVT use for dizziness presentations.

**Methods:**

We conducted a cohort study of all patients admitted with acute dizziness at Stavanger University Hospital from 2016 to 2022. Trends in admissions, triage, bedside clinical assessment, and intravenous thrombolysis (IVT) use were analyzed. Stroke mimics were defined as patients thrombolysed but subsequently discharged with a non-stroke diagnosis after MRI and neurovascular reassessment.

**Results:**

A total of 4,594 patients were admitted with acute dizziness, representing 1.9% of all ED admissions. Annual admissions increased from 455 in 2016 to 819 in 2022 (80%), with neurology admissions rising from 33 to 50%. IVT use in isolated dizziness almost doubled (17 in 2016 vs. 32 in 2022), peaking in 2020–2021 (>55 annually), while confirmed stroke cases remained stable (3–7/year). Stroke mimics accounted for most IVT-treated dizziness cases, exceeding 80% in patients with NIHSS 0. On average only 50% of thrombolysed patients had a complete neuro-ophthalmological examination.

**Conclusion:**

ED admissions for acute dizziness are rising, with increased IVT use but stable stroke incidence, leading to higher rates of stroke mimics. Incomplete bedside examinations and emphasis on faster DNT may contribute to misclassification. Standardized dizziness protocols, mandatory use of HINTS by trained clinicians, and stronger cross-specialty collaboration are needed to improve diagnostic accuracy while preserving timely stroke treatment.

## Introduction

Acute dizziness is a common and increasing presenting symptom in emergency departments (ED), accounting for approximately 4% of ED visits, with a lifetime incidence of 15–20% in the general population (1–3). Although most causes are benign, 3–5% of cases are due to posterior circulation stroke, which requires treatment and poses a significant diagnostic challenge (4–7). Up to 20% of posterior circulation strokes present with isolated dizziness, making accurate identification critical to avoid adverse outcomes, as these strokes are associated with high mortality if untreated (8, 9). Despite efforts to improve stroke treatment, challenges remain.

Posterior circulation strokes are often under- and misdiagnosed (“stroke chameleons”), adding to the complexity (10–12). While IVT in stroke mimics is generally safe, about 1% suffer symptomatic intracranial hemorrhage (13). Both stroke mimics and chameleons contribute to unnecessary costs and healthcare burden (14, 15).

CT and MRI have low sensitivity in detecting stroke among patients presenting with dizziness (16). Thus, clinical evaluation remains essential. Bedside tests, such as “Head Impulse, Nystagmus, and Test of Skew” (HINTS) and “SponTAneous Nystagmus, Direction, head Impulse test” (STANDING), have high diagnostic accuracy when performed by trained providers (17, 18).

Although there is a well-documented high diagnostic accuracy using HINTS and STANDING, little is known in terms of real-world application in acute stroke diagnosis; a fast-paced, high pressure setting with increasing focus on low door-to-needle times (DNT).

At Stavanger University Hospital, a simulation-based stroke project led to a marked reduction in door-to-needle-times (DNT) and improved patient outcomes (19). As part of ongoing quality assurance within this initiative, an increase in IVT use was identified, particularly among patients presenting with dizziness, a frequent stroke mimic. This observation prompted further investigation into trends, diagnostic practices and potential contributing factors in this patient group (20).

This study aims to evaluate factors contributing to misdiagnosis by analyzing (i) temporal trends in total admissions and triage (ii) quality of clinical assessments and (iii) patterns in IVT use for dizziness presentations.

These findings could guide quality improvement efforts aimed at balancing timely treatment with diagnostic precision.

## Materials and methods

### Study design and patient population

We conducted a prospective cohort study including all patients admitted to the ED at Stavanger University Hospital between January 2016 and December 2022. Temporal trends were analyzed for all ED admissions, while analysis of clinical quality and IVT patterns was assessed in patients treated with IVT.

All patients presenting with dizziness as the primary admitting complaint were retrospectively identified using keyword searches for “dizziness, vertigo, stroke” as well as admission diagnosis from ICPC-2 in patients referred by primary care practitioners. Patients included had one of the following discharge diagnoses from ICD-10 (R42, H81.0–H81.9, I61, I63, and I64).

### Emergency department structure

Norwegian hospitals operate with a centralized ED system, where all patients require a referral for admission. Referrals are made by general practitioners, out of hour’s primary care physicians, or paramedics. The ED at Stavanger University hospital is staffed 24/7 by on-call specialists in internal medicine, surgery, and neurology. Patients are typically evaluated by the department indicated in their referral, although cross-departmental consultations, particularly for patients with dizziness, are common. Referring physicians who direct patients to the Department of Neurology typically consult with on-call neurologist before admission to the ED.

### Patient assessment

#### Radiological evaluation

All patients treated with IVT underwent non-contrast CT, CT-angiography, and CT-perfusion imaging upon admission. In cases of wake-up stroke or unknown symptom onset, MRI was used as primary imaging modality. A follow-up MRI was routinely performed, typically within the 72 h after admission. Although initial MRI could be performed in selected cases based on clinical judgment, it was not routinely used as the primary imaging modality in patients presenting with dizziness.

#### Clinical evaluation

All stroke patients are primarily assessed by on-call neurology registrars in the emergency department. Neurological impairment was assessed using NIHSS and relevant neuro-ophthalmological tests, in particular HINTS. NIHSS was performed at admission, 2 and 24 h after IVT administration, and repeated on the day of discharge. For stroke patients, clinical evaluations are performed before radiological examinations. Although HINTS was part of the standard neurological examination procedures, specific training in its use was not systematically included in onboarding of new physicians.

### Data collection

Data for this study were obtained from the electronic patient record system (DIPS Arena) and the local IVT registry at Stavanger University Hospital. All patients with suspected acute ischaemic stroke who received IVT during the study period were prospectively included in the registry. The dataset encompassed demographic information, cerebrovascular risk factors, NIHSS scores at admission and 24 h, stroke mimic status (defined as thrombolysed patients subsequently discharged with a non-stroke diagnosis), in-hospital and 90d mortality, and modified Rankin Scale (mRS) scores at baseline and 3 months.

Key time points - including symptom onset, ED arrival, and IVT administration - were systematically recorded. Data were collected using a standardized form completed jointly by stroke nurses and neurology registrars, supplemented by documentation in the electronic medical record (EMR). A dedicated stroke research nurse is responsible for overseeing data completeness and quality. Collected data of NIHSS and HINTS are based on documentation in EMR.

### Classification of variables

Information collected included demographics (age, sex), clinical presentation (symptoms, NIHSS, HINTS), radiological findings (CT, MRI results), medical treatment (IVT administration, timing) and final diagnosis (stroke, stroke mimics, or other).

### Key variables include

**Figure 1:**
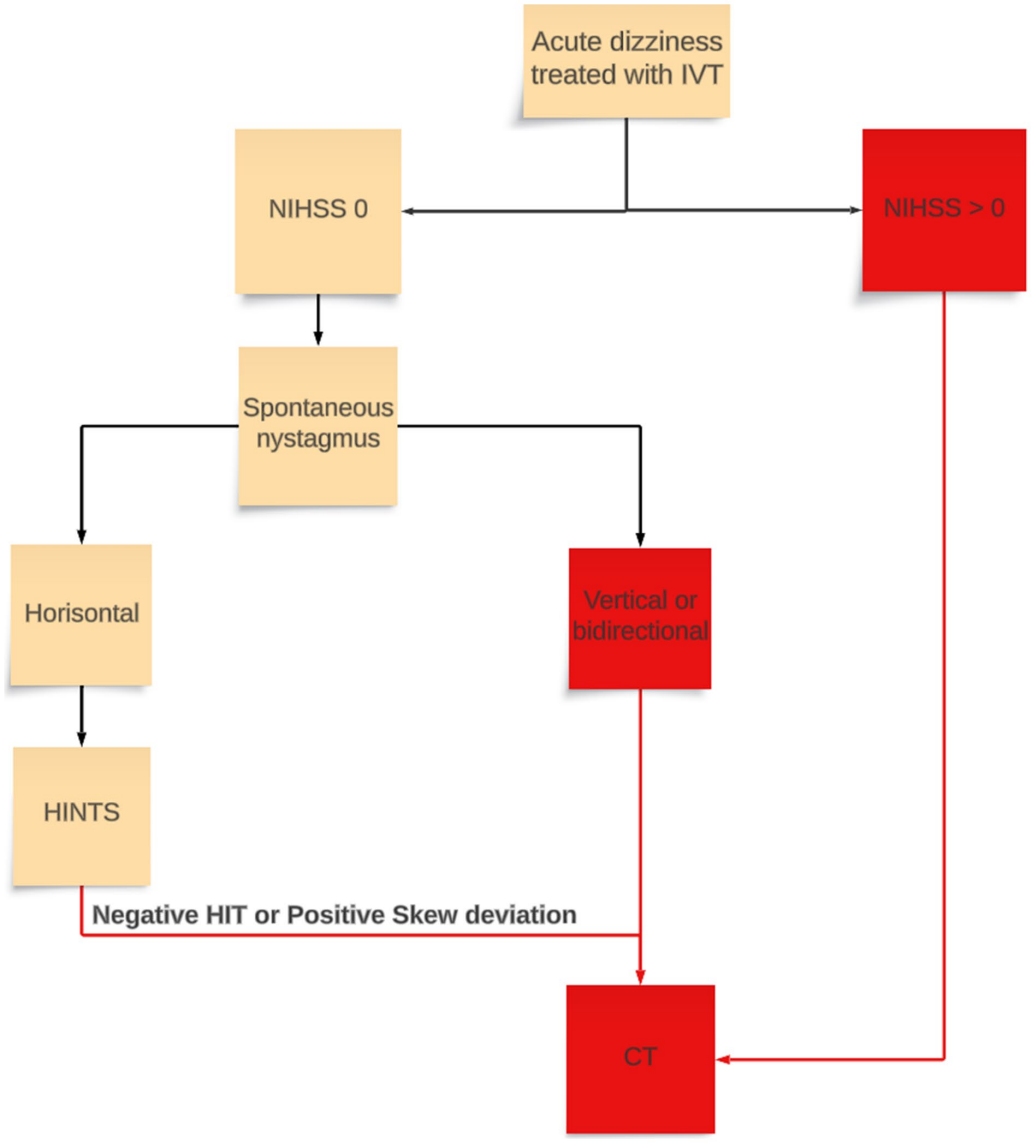
Flow chart for guideline clinical assessment of acute vertigo used for retrospective analysis. Red boxes and lines are where patient progresses to CT-brain. IVT, intravenous thrombolysis; NIHSS, National institute of health stroke scale; HIT, Head-impulse test; HINTS, Head-impulse nystagmus test of skew.

- Confirmed Strokes: Based on MRI findings and clinical assessment by neurologists.
- Stroke Mimics: Patients treated with IVT but retrospectively identified as non-stroke cases based on negative MRI and reassessment by the neurovascular team.
- Dizziness Diagnoses: Classified according to ICD-10 codes as peripheral (H81.1, H81.2, H81.8) or non-specific (R42, H81.3, H81.9) dizziness. Department admitted: Patients admitted to the Department of Internal Medicine or the Department of Ear, Nose and Throat with chief symptoms of dizziness, were also classified according to the ICD-classification into specific and non-specific dizziness diagnoses.
- Determination of correct acute dizziness stroke evaluation: To evaluate whether patients with acute dizziness treated with IVT where correctly examined, we developed an evaluation flow chart and used this for retrospective analysis of electronic medical records. The chart was revised by senior stroke neurologists within the research group. IVT-treated patients were divided into two groups based on NIHSS; NIHSS 0 and NIHSS >0. For patients with NIHSS >0, direct transport to emergent CT-brain would be determined as correct after NIHSS evaluation. For patients with acute vestibular syndrome and NIHSS 0, an otoneurologic evaluation including HINTS prior to CT-scan was deemed correct. Furthermore, if the correct examinations were performed, but were incorrectly interpreted, it was classified as incorrect (i.e., clear signs of vestibular neuritis or benign paroxysmal positional vertigo, but still progress to CT and administer IVT) (Figure 1).

### Bias minimization

To ensure consistency and reduce risk of misclassification, all final diagnoses in IVT-treated dizziness patients were retrospectively reassessed by a neurovascular team consisting of two consultants and one registrar. Additionally, all patients with NIHSS <5 and negative imaging were systematically reviewed for potential misclassification. Potentially misdiagnosed stroke patients were evaluated by all three members of review team to determine whether they should be classified as stroke mimics.

### Statistics

Statistical analyses were performed using SPSS Statistics version 24 (IBM Cooperation, Armonk, NY, United States). Categorical variables were summarized as frequencies and percentages, and continuous variables as means with standard deviations or medians with interquartile ranges, as appropriate. Comparisons of proportions of neurological referrals and admissions compared to total referrals and admissions in the emergency department were performed using chi-square tests or Fisher’s exact test where cell counts were small. For trends over time we used Cochran-Armitage test. Differences in continuous variables were evaluated using t-tests or Mann–Whitney U tests depending on data distribution. A *p*-value <0.05 was considered statistically significant.

### Ethics

The study was approved by the Regional Committee for Medical and Health Research Ethics (ID 23155, No. 2018/1895), the Norwegian Center for Research Data, and the local hospital authorities.

## Results

### Temporal trends in admissions for acute dizziness

**Figure 2:**
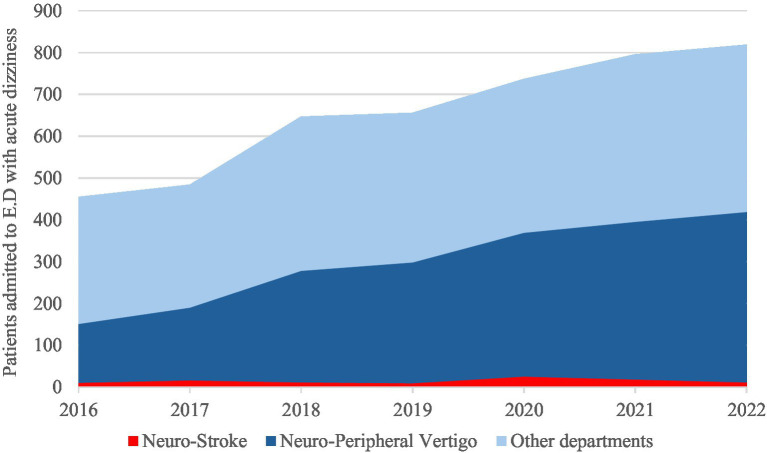
Temporal trends in emergency department admissions for acute dizziness at Stavanger University Hospital, 2016-2022. The stacked area chart shows the annual number of patients admitted with acute dizziness, stratified by admission department: neurology-stroke, neurology-peripheral vertigo, and other departments.

Between 2016 and 2022, a total of 4,594 patients were admitted to the ED at Stavanger University Hospital with a diagnosis of acute dizziness, representing 1.93% of all ED admissions during this period. The annual number of ED admissions for acute dizziness increased by 80%, from 455 cases in 2016 to 819 in 2022 (*p* < 0.001). There was also a notable shift in triage patterns: the proportion of patients with acute dizziness admitted to the Department of Neurology increasing from 151/455(33.2%) in 2016 to 408/819(49.9%) in 2022 (*p* < 0.001; Figure 2).

### Quality of clinical examination

**Figure 3:**
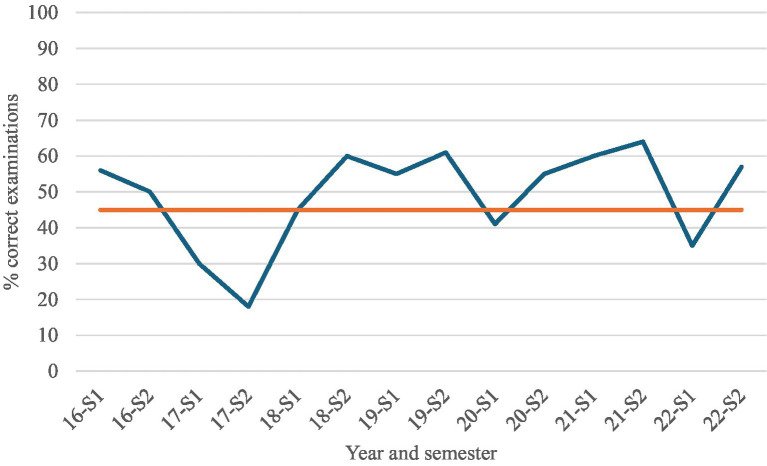
Temporal variation in guideline-adherent clinical examination among IVT-treated patients with acute dizziness, 2016-2022. Line graph showing the proportion of patients who underwent complete and guideline-appropriate bedside examination in the emergency department, stratified by semester (S1-S2).

We found inconsistencies in adherence to recommended diagnostic protocols (Figure 1). A median of 54.5%, (mean 50%) of patients treated with IVT for acute dizziness did not undergo a complete and appropriate sequence of clinical examinations in the ED between 2016 and 2022 (Figure 3). A majority of the incorrectly examined patients lacked documentation of HINTS examinations in EMR.

### Diagnostic practices

**Figure 4:**
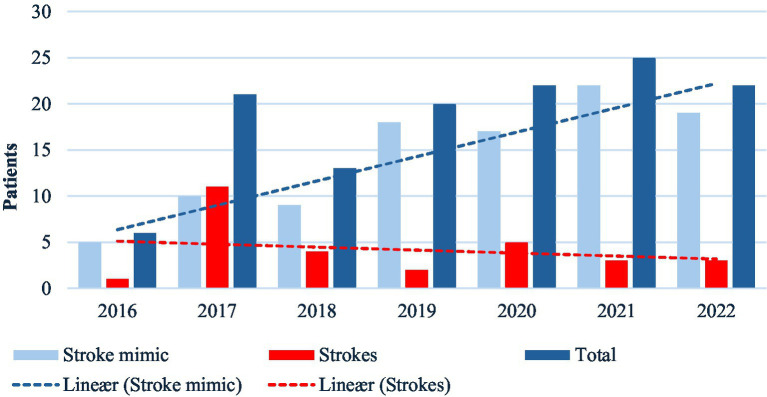
Temporal trends in intravenous thrombolysis among patients with acute dizziness, 2016-2022. Grouped bar chart showing annual number of IVT-treated patients categorized as confirmed stroke or stroke mimic. Bars represent absolute counts per year; dashed lines indicate linear trends. Increase in IVT use over time is predominantly driven by stroke mimics.

A total of 250 patients with acute dizziness received IVT between 2016 and 2022. One hundred twenty-three patients had isolated dizziness (NIHSS 0) (Table 1). The number of patients with isolated dizziness receiving IVT increased significantly from 17 cases in 2016 to 32 in 2022 (*p* = 0.0032), with peak years 2020 and 2021, with 58 and 56 patients treated, respectively. Despite the increased use of IVT for acute dizziness, the number of confirmed cases of stroke remained stable (3–7 cases/year), while the proportion of stroke mimics among IVT-treated patients with acute dizziness steadily increased across all NIHSS score (Figure 4).

**Table 1:** Patient demographics, clinical outcomes and safety variables stratified by admission NIHSS score (NIHSS = 0 vs. NIHSS > 0) and final diagnosis (stroke vs. stroke mimic).

Variable	NIHSS = 0 (*n* = 123)		NIHSS > 0 (*n* = 127)	
	Stroke (*n* = 28)	Mimic (*n* = 95)	Stroke (*n* = 73)	Mimic (*n* = 54)
Demographics				
Age	69	61	72	59
Sex (f)	46,4	53,7	41,1	61,1
Outcome				
NIHSS admission Mdn	–	–	2	2
NIHSS 24 h Mdn	0	0	0	0
EVT	0	0	3	0
90d mRS Mdn	0	0	1	0
90d mRS IQR	0–1	0–1	0–2	0–1
90d mortality	0	0	0	0
Safety				
sICH	0	0	1	0
aICH	1	3	7	1
Other hemorrhages	0	0	1	0
Angiooedema	0	0	1	1

NIHSS, National institute of health stroke scale; EVT, endovascular therapy; mRS, modified rankin scale; Mdn, median; IQR, interquartile ranges; sICH, symptomatic intracranial hemorrhage; aICH, asymptomatic intracranial hemorrhage.

**Figure 5:**
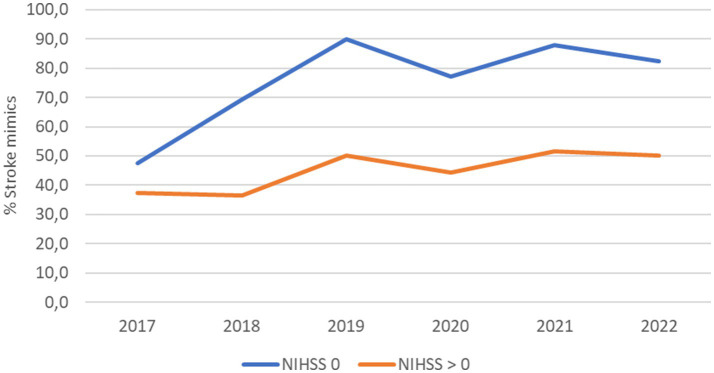
Proportion of stroke mimics among patients presenting with acute dizziness stratified by NIHSS score 2017-2022. Time-series line plot showing the percentage of patients classified as stroke mimics among those with isolated dizziness (NIHSS = 0) and those with dizziness plus neurological deficits (NIHSS > 0).

When patients were categorized by NIHSS score, a significant increase in stroke mimics was particularly evident in the isolated dizziness group (NIHSS 0). The proportion of stroke mimics in this group exceeded 80% in the latter years of the study (Figure 5).

**Figure 6:**
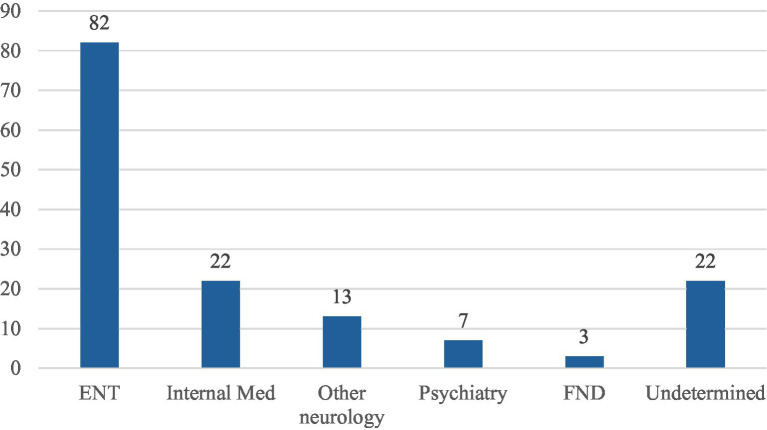
Stroke mimic categories. ENT, ear-nose-throat peripheral vertigo. Internal medical causes most commonly orthostatic hypotension, syncope, arrythmia, and infections. Other neurology mostly migraine and epilepsy. Psychiatry primarily anxiety attacks. FND, functional neurologic disorder. Undetermined for patients not clearly diagnosed with one of the previous categories.

Stroke mimics were most likely to have peripheral vertigo from ENT diagnosis, followed by internal medical illnesses and other neurological disorders like migraine and epilepsy (Figure 6).

One patient in the NIHSS >0 stroke group underwent a symptomatic intracranial hemorrhage (sICH). In the mimic groups there were no accounts of sICH, and only four accounts of asymptomatic intracranial hemorrhage (aICH) which amounts to 2.68%.

## Discussion

We identified a significant increase in all ED admissions for acute dizziness between 2016 and 2022 at Stavanger University Hospital. Especially the department of neurology had a 170,2% increase in acute dizziness admissions. This was paralleled by a rise in IVT treated patients subsequently diagnosed as. Despite the increase in IVT use, the number of confirmed strokes with isolated dizziness remained stable, suggesting suboptimal diagnostic accuracy. One contributing factor may be that only about half of the patients treated with IVT underwent a complete and guideline-appropriate clinical assessment in the ED.

Acute dizziness is a frequent and diagnostically challenging presentation in the ED. Distinguishing benign peripheral vestibular disorders from life-threatening causes such as posterior circulation stroke continues to be a major clinical challenge. Our findings reinforce the diagnostic difficulty inherent to acute dizziness. Patients with acute dizziness are at risk of both undertreatment and overtreatment, and misdiagnosis may lead to delays in stroke care, inappropriate use of thrombolysis, and avoidable resource utilization (15, 21). Prior studies report that up to 35% of posterior circulation strokes presenting with dizziness are initially missed (1, 22). In addition, dizziness accounts for approximately 25% of all stroke mimics encountered in the emergency department (23). However, the proportion of stroke mimics among patients receiving IVT for isolated acute dizziness has not been previously reported according to our knowledge. In our center, stroke mimics comprised up to 80% of IVT-treated isolated dizziness patients in the later years of the study, suggesting that isolated dizziness is both an important stroke mimic phenotype and a common trigger for potentially unnecessary thrombolysis.

These diagnostic uncertainties influence referral patterns and resource use. In 2022, nearly half of all patients presenting with acute dizziness were admitted for neurological evaluation in the ED. This pattern suggests a tendency to overattribute dizziness to neurological causes in our population. For comparison, one US study found that almost 50% of ED dizziness presentations had medical causes, 33% were otologic or vestibular in origin, and only 11% were neurological (5). Such triaging behavior likely reflects clinicians’ concern that dizziness could represent a posterior circulation stroke and may contribute to rising admissions and overtreatment with IVT in otherwise low-risk patients (1).

System-level changes may also have influenced decision-making. During the study period, focused efforts to reduce door-to-needle times likely prioritized rapid intervention. Previous studies have shown that accelerating treatment pathways may decrease diagnostic specificity and increase mimic treatment (19, 20, 24). Limited use of bedside assessments, such as the HINTS exam which has demonstrated high diagnostic accuracy, may have further contributed (25, 26). The combination of rising dizziness presentations, stable stroke incidence, frequent mimic treatment and incomplete bedside assessment identifies clear, modifiable contributors within the care pathway. These contributors represent clear and actionable targets for improvement that could substantially reduce diagnostic errors and inappropriate thrombolysis in patients presenting with dizziness.

## Conclusion

This study demonstrates a substantial rise in dizziness-related ED admissions and IVT use over the past 7 years without a corresponding increase in confirmed posterior circulation stroke. The high proportion of stroke mimics among IVT-treated patients with dizziness highlights significant diagnostic uncertainty and indicates that current assessment pathways may favor rapid treatment over clinical accuracy.

To address these challenges, we propose a multifaceted strategy consisting of: (i) implementation of a standardized diagnostic protocol for acute dizziness, (ii) mandatory use of bedside assessment tools such as HINTS performed by trained clinicians, and (iii) decision-support aids to guide triage and treatment decisions in the ED. Strengthened collaboration between neurology, emergency medicine and ENT, supported by targeted education and simulation-based training, may further enhance diagnostic confidence and consistency.

Adoption of these measures has the potential to reduce unnecessary thrombolysis, optimize resource utilization and improve patient safety, ensuring that rapid access to stroke therapy is preserved for those who truly benefit.

## Limitations

This study has several limitations. Although IVT-treated patients were prospectively captured in a local registry, the assessment of bedside clinical examination quality was based on retrospective review of electronic documentation and is therefore subject to documentation bias. The absence of routine video-oculography and adjudicated HINTS testing means that we could not confirm whether HINTS was performed but not documented, nor independently evaluate diagnostic accuracy at the bedside.

Identification of dizziness presentations relied on diagnostic coding and keyword searches, which may have resulted in misclassification or missed cases, and early MRI can fail to detect posterior circulation infarction, potentially leading to underestimation of true stroke incidence. Some relevant clinical variables, such as symptom duration prior to arrival or history of recurrent dizziness, were unavailable and could not be assessed.

As a single-center study, findings may not be generalisable to hospitals with different referral pathways, staffing patterns or imaging access. Secular changes in staffing, workflow or referral behavior over the seven-year period may have influenced observed trends but could not be directly measured. However, Stavanger University Hospital is the sole acute care provider for the region, and the inclusion of all consecutive dizziness presentations and IVT-treated cases provides population-level completeness and strengthens internal validity.

## Data Availability

The raw data supporting the conclusions of this article will be made available by the authors, without undue reservation.

## References

[1] Kerber KA Brown DL Lisabeth LD Smith MA Morgenstern LB. Stroke among patients with dizziness, vertigo, and imbalance in the emergency department: a population-based study. Stroke. (2006) 37:2484–7. doi: 10.1161/01.STR.0000240329.48263.0d, 16946161 PMC1779945

[2] Neuhauser HK. The epidemiology of dizziness and vertigo. Handb Clin Neurol. (2016) 137:67–82. doi: 10.1016/B978-0-444-63437-5.00005-4, 27638063

[3] Goplen FK Wiik R. Patients admitted to hospital for vestibular neuritis in 2011-2021. Tidsskr Nor Legeforen. (2023) 143:80. doi: 10.4045/tidsskr.23.0080, 37830970

[4] Saber Tehrani AS Kattah JC Kerber KA Gold DR Zee DS Urrutia VC . Diagnosing Stroke in Acute Dizziness and Vertigo: Pitfalls and Pearls. Stroke. (2018) 49:788–95. doi: 10.1161/STROKEAHA.117.016979, 29459396 PMC5829023

[5] Newman-Toker DE Hsieh YH Camargo CA Pelletier AJ Butchy GT Edlow JA. Spectrum of dizziness visits to US emergency departments: cross-sectional analysis from a nationally representative sample. Mayo Clin Proc. (2008) 83:765–75. doi: 10.4065/83.7.765, 18613993 PMC3536475

[6] Cheung CS Mak PS Manley KV Lam JM Tsang AY Chan HM . Predictors of important neurological causes of dizziness among patients presenting to the emergency department. Emerg Med J. (2010) 27:517–21. doi: 10.1136/emj.2009.078014, 20584952

[7] Lee DH Kim WY Shim BS Kim TS Ahn JH Chung JW . Characteristics of central lesions in patients with dizziness determined by diffusion MRI in the emergency department. Emerg Med J. (2014) 31:641–4. doi: 10.1136/emermed-2013-202674, 23722117

[8] Venhovens J Meulstee J Verhagen WI. Acute vestibular syndrome: a critical review and diagnostic algorithm concerning the clinical differentiation of peripheral versus central aetiologies in the emergency department. J Neurol. (2016) 263:2151–7. doi: 10.1007/s00415-016-8081-8, 26984607

[9] Doijiri R Uno H Miyashita K Ihara M Nagatsuka K. How commonly is stroke found in patients with isolated Vertigo or dizziness attack? J Stroke Cerebrovasc Dis. (2016) 25:2549–52. doi: 10.1016/j.jstrokecerebrovasdis.2016.06.038, 27495834

[10] Gurley KL Edlow JA. Avoiding misdiagnosis in patients with posterior circulation ischemia: a narrative review. Acad Emerg Med. (2019) 26:1273–84. doi: 10.1111/acem.13830, 31295763

[11] Tu LH Mahajan A Minja FJ Navaratnam D Melnick ER. Pilot MRI-based strategies to improve the detection of stroke in patients with dizziness/vertigo. Clin Imaging. (2022) 82:234–6. doi: 10.1016/j.clinimag.2021.12.001, 34902799

[12] Arch AE Weisman DC Coca S Nystrom KV Wira CR Schindler JL. Missed ischemic stroke diagnosis in the emergency department by emergency medicine and neurology services. Stroke. (2016) 47:668–73. doi: 10.1161/STROKEAHA.115.010613, 26846858

[13] Zinkstok SM Engelter ST Gensicke H Lyrer PA Ringleb PA Artto V . Safety of thrombolysis in stroke mimics: results from a multicenter cohort study. Stroke. (2013) 44:1080–4. doi: 10.1161/STROKEAHA.111.000126, 23444310

[14] Buck BH Akhtar N Alrohimi A Khan K Shuaib A. Stroke mimics: incidence, aetiology, clinical features and treatment. Ann Med. (2021) 53:420–36. doi: 10.1080/07853890.2021.1890205, 33678099 PMC7939567

[15] Goyal N Male S al Wafai A Bellamkonda S Zand R. Cost burden of stroke mimics and transient ischemic attack after intravenous tissue plasminogen activator treatment. J Stroke Cerebrovasc Dis. (2015) 24:828–33. doi: 10.1016/j.jstrokecerebrovasdis.2014.11.023, 25735708

[16] Schneider AM Neuhaus AA Hadley G Balami JS Harston GW DeLuca G . Posterior circulation ischaemic stroke diagnosis and management. Clin Med (Lond). (2023) 23:219–27. doi: 10.7861/clinmed.2022-0499, 37236792 PMC11046504

[17] Gerlier C Hoarau M Fels A Vitaux H Mousset C Farhat W . Differentiating central from peripheral causes of acute vertigo in an emergency setting with the HINTS, STANDING, and ABCD2 tests: a diagnostic cohort study. Acad Emerg Med. (2021) 28:1368–78. doi: 10.1111/acem.14337, 34245635

[18] Gottlieb M Peksa GD Carlson JN. Head impulse, nystagmus, and test of skew examination for diagnosing central causes of acute vestibular syndrome. Cochrane Database Syst Rev. (2023) 2023:CD015089. doi: 10.1002/14651858.CD015089.pub2, 37916744 PMC10620998

[19] Ajmi SC Advani R Fjetland L Kurz KD Lindner T Qvindesland SA . Reducing door-to-needle times in stroke thrombolysis to 13 min through protocol revision and simulation training: a quality improvement project in a Norwegian stroke Centre. BMJ Qual Saf. (2019) 28:939–48. doi: 10.1136/bmjqs-2018-009117, 31256015

[20] Hollesli LJ Ajmi SC Kurz MW Tysland TB Hagir M Dalen I . Simulation-based team-training in acute stroke: is it safe to speed up? Brain Behav. (2022) 12:e2814. doi: 10.1002/brb3.2814, 36416494 PMC9759129

[21] Tarnutzer AA Koohi N Lee SU Kaski D. Diagnostic errors in the acutely dizzy patient-lessons learned. Brain Sci. (2025) 15:55. doi: 10.3390/brainsci15010055, 39851423 PMC11764146

[22] Krishnan K Bassilious K Eriksen E Bath PM Sprigg N Brækken SK . Posterior circulation stroke diagnosis using HINTS in patients presenting with acute vestibular syndrome: a systematic review. Eur Stroke J. (2019) 4:233–9. doi: 10.1177/2396987319843701, 31984230 PMC6960692

[23] Pohl M Hesszenberger D Kapus K Meszaros J Feher A Varadi I . Ischemic stroke mimics: a comprehensive review. J Clin Neurosci. (2021) 93:174–82. doi: 10.1016/j.jocn.2021.09.025, 34656244

[24] Liberman AL Liotta EM Caprio FZ Ruff I Maas MB Bernstein RA . Do efforts to decrease door-to-needle time risk increasing stroke mimic treatment rates? Neurol Clin Pract. (2015) 5:247–52. doi: 10.1212/CPJ.0000000000000122, 26124982 PMC4469347

[25] Kattah JC Talkad AV Wang DZ Hsieh YH Newman-Toker DE. HINTS to diagnose stroke in the acute vestibular syndrome: three-step bedside oculomotor examination more sensitive than early MRI diffusion-weighted imaging. Stroke. (2009) 40:3504–10. doi: 10.1161/STROKEAHA.109.551234, 19762709 PMC4593511

[26] Newman-Toker DE Curthoys IS Halmagyi GM. Diagnosing stroke in acute Vertigo: the HINTS family of eye movement tests and the future of the "eye ECG". Semin Neurol. (2015) 35:506–21. doi: 10.1055/s-0035-1564298, 26444396 PMC9122512

